# Oral health in chronic obstructive pulmonary disease: are physicians prepared to integrate it into routine care?

**DOI:** 10.3389/fmed.2026.1945808

**Published:** 2026-09-16

**Authors:** Rayan A. Siraj, Aminah S. Mengash, Maryam M. Almulhem, Munerah N. Almulhem, Anabelle P. Buran-Omar, Abdulelah M. Aldhahir, Abdullah A. Alqarni, Jaber S. Alqahtani, Saleh S. Algarni, Abdulrhman S. Alghamdi, Ahmed H. Alasimi, Mohammed M. Alyami, Faisal F. Alotaibi, Mohammed D. Alelyani

**Affiliations:** 1 Department of Respiratory Therapy, College of Applied Medical Sciences, King Faisal University, Al-Ahsa, Saudi Arabia; 2 Department of Public Health, College of Applied Medical Sciences, King Faisal University, Al-Ahsa, Saudi Arabia; 3 Department of Nursing, College of Applied Medical Sciences, King Faisal University, Al-Ahsa, Saudi Arabia; 4 Respiratory Therapy Program, Department of Nursing, College of Nursing and Health Sciences, Jazan University, Jazan, Saudi Arabia; 5 Department of Respiratory Therapy, Faculty of Medical Rehabilitation Sciences, King Abdulaziz University, Jeddah, Saudi Arabia; 6 Respiratory Therapy Unit, King Abdulaziz University Hospital, Jeddah, Saudi Arabia; 7 Department of Respiratory Care, Prince Sultan Military College of Health Sciences, Dammam, Saudi Arabia; 8 Department of Respiratory Therapy, College of Applied Medical Sciences, King Saud bin Abdulaziz University for Health Sciences, Riyadh, Saudi Arabia; 9 King Abdullah International Medical Research Centre, Riyadh, Saudi Arabia; 10 Department of Rehabilitation Health Sciences, College of Applied Medical Sciences, King Saud University, Riyadh, Saudi Arabia; 11 National Heart and Lung Institute, Imperial College London, London, United Kingdom; 12 Respiratory Therapy Department, Batterjee Medical College, Khamis Mushait, Saudi Arabia; 13 Ministry of Interior, Public Security, Medical Service, Dhahran, Saudi Arabia; 14 Department of Dentistry, Ministry of Interior, Public Security, Medical Service, Dhahran, Saudi Arabia

**Keywords:** chronic obstructive pulmonary disease, comorbidity, oral health, physicians, Saudi Arabia

## Abstract

**Background:**

Poor oral health has been associated with adverse outcomes in chronic obstructive pulmonary disease (COPD), yet its integration into routine COPD care remains poorly understood. This study evaluated physicians’ attitudes, confidence, clinical practices, perceived barriers, and factors associated with routine oral health assessment.

**Methods:**

A national cross-sectional survey was conducted among general practitioners, pulmonologists, and critical care physicians practising in Saudi Arabia between October 2025 and April 2026. The questionnaire assessed physicians’ attitudes, confidence, oral health practices, and perceived barriers related to oral health in COPD. Composite attitude and confidence scores were calculated by averaging responses across the corresponding Likert-scale items. Univariable and multivariable logistic regression analyses were performed to identify factors associated with routine oral health assessment.

**Results:**

A total of 1,095 physicians completed the survey. Routine oral health assessment was infrequent, with only 24.7% reporting that they often or always assessed oral health during COPD care, despite generally neutral to moderately favourable attitudes towards oral health in COPD. Among physicians who assessed oral health at least sometimes, documentation of oral health findings was the most common management strategy (45.2%). The principal barriers were lack of formal training (38.5%), insufficient time (28.1%), uncertainty regarding professional responsibility (24.1%), and the absence of workplace protocols (22.6%). In the multivariable analysis, pulmonologists were more likely than General Practitioners to routinely assess oral health (adjusted OR 1.65, 95% CI 1.11–2.45; *P* = 0.013). Higher attitude (adjusted OR 1.31, 95% CI 1.04–1.64; *P* = 0.022) and confidence scores (adjusted OR 3.00, 95% CI 2.26–3.99; *P* < 0.001) were independently associated with routine oral health assessment.

**Conclusion:**

Despite generally positive attitudes towards oral health in COPD, routine oral health assessment remains infrequently incorporated into clinical practice. The identified associations highlight potential areas for improving the integration of oral health into COPD care; however, the cross-sectional design does not permit causal inference.

## 1 Introduction

Chronic obstructive pulmonary disease (COPD) is a leading cause of morbidity and mortality worldwide and represents a substantial burden on health systems (1). Although characterised by persistent airflow limitation and chronic airway inflammation, COPD is now recognised as a complex multisystem disease that frequently coexists with cardiovascular disease, diabetes, osteoporosis, depression, and other comorbidities that substantially influence symptom burden, exacerbation frequency, quality of life, and survival (2). Despite growing recognition of the importance of comorbidities, clinical management and research have remained largely focused on the respiratory manifestations of COPD, while many non-respiratory conditions continue to receive comparatively little attention (3). Oral health is one such neglected aspect of COPD care. Conditions including periodontitis, dental caries, xerostomia, and poor oral hygiene are common among patients with COPD but are rarely assessed during routine respiratory care (4).

A recent meta-analysis of 41 studies reported a pooled prevalence of periodontitis of approximately 35% among patients with COPD, substantially higher than in individuals without COPD (5). Patients with COPD also exhibited greater probing depth and clinical attachment loss, indicating more severe periodontal disease. Furthermore, a meta-analysis of observational studies found that periodontal disease was associated with more than a two-fold increase in the odds of COPD (6). Collectively, these findings suggest that periodontal disease is not only a common comorbidity in COPD but may also contribute to disease progression and adverse clinical outcomes.

Several mechanisms may explain this association (7). Periodontal disease promotes chronic systemic inflammation that may amplify the inflammatory processes characteristic of COPD. In addition, periodontal pockets and subgingival biofilms can act as reservoirs for respiratory pathogens that may be aspirated into the lower airways, increasing susceptibility to respiratory infection (8, 9). These mechanisms may contribute to disease progression and help explain the higher rates of exacerbations and hospitalisations reported among patients with poor oral health (10). Poor oral health may also impair nutritional status through tooth loss, impaired mastication, and oral pain, leading to reduced dietary intake, while poorer periodontal health has been associated with lower lean body mass and serum albumin concentrations (11).

Physicians involved in the long-term management of COPD are well positioned to identify oral health problems during routine clinical encounters through simple measures such as oral inspection, enquiring about oral hygiene practices, documenting oral health findings, and facilitating referral to dental services. Although the Global Initiative for Chronic Obstructive Lung Disease (GOLD) recommends systematic identification and management of comorbidities as part of routine COPD care (12), it provides little guidance on oral health assessment, including responsibility for assessment, implementation, or referral pathways. Consequently, oral health assessment remains poorly integrated into COPD care, with inadequate training, limited confidence, time constraints, and the absence of clear referral pathways identified as important barriers (4).

Despite growing evidence linking poor oral health with adverse COPD outcomes, research examining healthcare professionals' management of oral health in COPD remains limited. Existing studies have focused predominantly on dentists, nurses, or mixed healthcare professional populations and have largely been restricted to evaluating knowledge or awareness (13). Studies specifically examining physicians' attitudes, confidence, routine clinical practices, perceived barriers, and factors associated with routine oral health assessment in COPD are scarce, particularly in the Middle East.

Therefore, this study aimed to evaluate physicians' attitudes, confidence, current clinical practices, and perceived barriers regarding oral health assessment and management in patients with COPD in Saudi Arabia, and to identify factors associated with routine oral health assessment. Identifying these gaps is an important first step towards developing educational interventions, structured clinical protocols, and referral pathways that support the integration of oral health into comprehensive COPD management.

## 2 Methods

### 2.1 Study design and participants

A national cross-sectional survey was conducted using an online questionnaire to examine physicians’ attitudes, confidence, clinical practices, and perceived barriers related to oral health assessment and management in patients with chronic obstructive pulmonary disease (COPD). The survey was administered via the SurveyMonkey platform between 1 October 2025 and 30 April 2026.

Eligible participants were physicians directly involved in the management of patients with COPD, including general practitioners, pulmonologists, and critical care physicians working in governmental and private healthcare institutions across Saudi Arabia. A convenience sampling strategy was adopted to recruit participants from different geographical regions of the country.

Before commencing the survey, physicians were presented with an information page outlining the study objectives, confidentiality of the collected data, and the voluntary nature of participation. Participants were informed that they could discontinue the survey at any stage without penalty. No personally identifiable information was collected. Electronic informed consent was obtained by requiring participants to answer “Yes” to the question, “Do you agree to participate in this study?” before accessing the questionnaire. Completion of the survey required approximately 7–10 min, and the survey platform settings were configured to minimise duplicate submissions. All questionnaire items were mandatory, and participants were able to review and modify their responses before final submission.

To enhance nationwide participation, the survey was disseminated through professional societies, physician networks, and widely used social media platforms, including X (formerly Twitter), WhatsApp, and Telegram. These distribution methods facilitated recruitment of physicians from governmental and private healthcare institutions across all administrative regions of Saudi Arabia. Because the survey was disseminated through multiple professional networks and social media platforms, and recipients could forward it, the total number of eligible physicians who received or viewed the survey invitation could not be reliably determined. Therefore, a conventional response rate could not be calculated.

Based on workforce statistics published by the Saudi Health Council, the target population comprised approximately 15,856 general practitioners, 727 pulmonologists, and 3,185 critical care physicians practising in Saudi Arabia (14). Because no previous national data describing physicians' oral health assessment practices in COPD were available, the required sample size was estimated using the single-population proportion formula with a conservative prevalence estimate of 50%, a 95% confidence level, and a 5% margin of error (15, 16). The minimum required sample size was 384 physicians. To accommodate incomplete responses, this number was increased by 20%. The final study included 1,095 physicians, substantially exceeding the calculated sample size.

### 2.2 Questionnaire

The questionnaire was designed using items adapted from previous survey-based studies investigating the management of COPD-related comorbidities (4, 7, 17) together with evidence identified through a review of the contemporary literature on oral health in COPD (18, 19). Following the initial development, the questionnaire was reviewed by a multidisciplinary panel comprising respiratory physicians, dentists, public health researchers, and experts in survey methodology to evaluate its content validity, clarity, and relevance to the study objectives. A pilot evaluation was subsequently undertaken with 20 physicians representing the target specialties to assess the readability, comprehensibility, and overall structure of the questionnaire. Comments received during the pilot phase informed minor revisions to the wording and presentation of several items before the questionnaire was finalised. Responses collected during the pilot study were not included in the final analyses.

The final questionnaire comprised five domains. The first domain collected demographic and professional information, including participants' age, gender, physician specialty, primary workplace, years of clinical experience, average monthly COPD caseload, and previous formal training related to oral health in COPD. Previous formal training was assessed using the question: “Have you received formal training on oral care in patients with COPD?” (Yes/No).

The second domain assessed physicians’ attitudes towards oral health in COPD using 10 statements rated on a five-point Likert scale ranging from Strongly disagree (1) to Strongly agree (5). Attitude was conceptualised as physicians’ beliefs and evaluations regarding the importance, relevance, and integration of oral health within COPD care. An overall attitude score was calculated as the mean of the 10 items. No items were reverse-scored, as agreement with negatively phrased statements reflected recognition of existing gaps in oral health integration rather than an unfavourable attitude towards oral health. Higher scores therefore reflected more favourable attitudes towards the importance and integration of oral health in COPD care. The third domain evaluated physicians’ confidence in recognising, discussing, documenting, and managing oral health issues in patients with COPD using eight statements rated on a five-point Likert scale ranging from Not confident (1) to Completely confident (5). Confidence was conceptualised as physicians’ perceived self-efficacy in performing oral health-related activities within COPD care, distinguishing it from attitudes reflecting physicians’ beliefs and evaluations regarding the importance and integration of oral health. A composite confidence score was calculated by averaging responses across the eight items, with higher scores reflecting greater confidence in oral health assessment and management.

The fourth domain explored current clinical practice, including the frequency of routine oral health assessment, enquiring about patients' oral hygiene practices, and collaboration with dental professionals. Responses were recorded using a five-point frequency scale ranging from Never to Always. Participants who reported assessing oral health sometimes, often, or always were subsequently asked to indicate the management strategies they typically adopted when poor oral health was identified. These included referral to dental professionals, provision of oral health education, documentation of oral health findings, monitoring over time, provision of direct oral care, and taking no specific action. Multiple responses were permitted for these management items.

The final domain examined perceived barriers to incorporating oral health into routine COPD care. Barrier items were developed based on previous survey-based studies (4, 7, 17) and the literature on oral health in COPD (18, 19) and were further reviewed by the multidisciplinary expert panel. The items included lack of formal training, insufficient time, uncertainty regarding professional responsibility, absence of workplace protocols, limited access to dental professionals, lack of confidence, unclear referral pathways, limited prioritisation of oral health in COPD-related continuing education, patients rarely reporting oral health problems, and insufficient resources. Participants were permitted to select more than one barrier.

The internal consistency of the attitude and confidence domains was evaluated using Cronbach's alpha coefficient. Both domains demonstrated acceptable internal consistency (Cronbach's *α* ≥ 0.70).

### 2.3 Ethical considerations

Ethical approval was obtained from the Research Ethics Committee of King Faisal University (Approval ID: KFU-REC-2025-SEP-ETHICS3589). The study was conducted in accordance with the ethical principles of the Declaration of Helsinki. Participation was voluntary, and all responses were collected anonymously. Participants provided electronic informed consent before commencing the questionnaire.

### 2.4 Statistical analysis

Data were analysed using Stata version 16.0 (StataCorp LP, College Station, TX, USA). Categorical variables were summarised as frequencies and percentages. For descriptive analyses of the attitude and confidence items, responses were presented across the five-point Likert scale. Composite attitude and confidence scores were calculated by averaging responses across the corresponding items, resulting in scores ranging from 1 to 5. No attitude items were reverse-scored, as described above. Higher scores indicated more favourable attitudes towards oral health in COPD and greater confidence in oral health assessment and management, respectively. The relationship between the composite attitude and confidence scores was examined using Pearson's correlation coefficient. Multicollinearity among variables included in the multivariable model was assessed using variance inflation factors (VIFs), with a VIF ≥5 considered indicative of potentially problematic multicollinearity Univariable logistic regression analyses were initially performed to examine the association between demographic and professional characteristics and physicians' self-reported routine oral health assessment. The primary outcome was derived from the questionnaire item assessing the frequency of routine oral health assessment during COPD care and was dichotomised as routine assessment (“Often” or “Always”) vs. non-routine assessment (“Never”, “Rarely”, or “Sometimes”). Independent variables included age group, gender, physician specialty, primary workplace, years of clinical experience, average monthly COPD caseload, previous formal training related to oral health in COPD, and the composite attitude and confidence scores. Variables included in the multivariable logistic regression model were selected *a priori* based on the study objectives, previous literature, and their plausible relationship with physicians’ oral health assessment practices, irrespective of their statistical significance in the univariable analyses. Odds ratios (ORs) with corresponding 95% confidence intervals (CIs) were reported, and statistical significance was defined as *P* < 0.05.

## 3 Results

### 3.1 Participant characteristics

A total of 1,095 physicians were included in the analysis. General practitioners constituted the largest specialty group (42.8%), followed by critical care physicians (33.3%) and pulmonologists (23.8%). Most participants worked in government hospitals (62.1%), had 6–10 years of clinical experience (36.2%), managed 10–30 patients with COPD per month (34.2%), and reported having received formal training on oral health in COPD (59.4%) (Table 1).

**Table 1:** Demographic and professional characteristics of physicians participating in the study (*N* = 1,095).

Characteristic	*n* (%)
Age group (years)	
24–30	274 (25.0)
31–35	455 (41.6)
36–40	238 (21.7)
>40	128 (11.7)
Gender	
Male	700 (63.9)
Female	395 (36.1)
Physician specialty	
Critical Care Physician	365 (33.3)
Pulmonologist	261 (23.8)
General Practitioner	469 (42.8)
Primary workplace	
Government hospital	680 (62.1)
Private hospital	415 (37.9)
Years of clinical experience	
Less than 1 year	152 (13.9)
1–5 years	303 (27.7)
6–10 years	396 (36.2)
More than 10 years	244 (22.3)
COPD patients managed per month	
<10	202 (18.4)
10–30	374 (34.2)
31–50	365 (33.3)
>50	154 (14.1)
Formal training on oral health in COPD	
Yes	650 (59.4)
No	445 (40.6)

Data are presented as *n* (%). Percentages may not total exactly 100% because of rounding.

COPD, chronic obstructive pulmonary disease; n, number.

### 3.2 Physicians' attitudes towards oral health in patients with COPD

Physicians demonstrated generally neutral to moderately favourable attitudes towards oral health in patients with COPD, with a mean attitude score of 2.89 ± 0.83 (range, 1–5). Across most statements, the largest proportion of participants selected “Neither agree nor disagree” (23.4%–29.2%), followed by “Agree” (17.7%–26.0%), indicating a tendency towards agreement rather than strong endorsement. Agreement was highest for the statement that oral health problems reduce quality of life in patients with COPD (36.5%), followed by the need for collaboration between medical and dental professionals (36.1%). In contrast, only 25.3% of physicians agreed or strongly agreed that oral health is a critical component of systemic health in individuals with COPD (Table 2).

**Table 2:** Attitudes toward oral health in patients with COPD among physicians.

Statement	Strongly disagree *n* (%)	Disagree *n* (%)	Neither agree nor disagree *n* (%)	Agree *n* (%)	Strongly agree *n* (%)
Oral health is a critical component of systemic health in individuals with COPD.	290 (26.5)	272 (24.8)	256 (23.4)	194 (17.7)	83 (7.6)
Oral health-related inflammation contributes to COPD progression.	175 (16.0)	243 (22.2)	320 (29.2)	249 (22.7)	108 (9.9)
Oral health deterioration increases bacterial colonization in the respiratory tract.	183 (16.7)	236 (21.6)	311 (28.4)	246 (22.5)	119 (10.9)
Oral health problems reduce the quality of life in patients with COPD.	148 (13.5)	232 (21.2)	315 (28.8)	253 (23.1)	147 (13.4)
Oral health is a modifiable risk factor in COPD care.	181 (16.5)	230 (21.0)	314 (28.7)	285 (26.0)	85 (7.8)
Oral health is frequently overlooked in COPD management.	199 (18.2)	228 (20.8)	273 (24.9)	270 (24.7)	125 (11.4)
Oral health is not addressed in most COPD training programs for healthcare providers.	178 (16.3)	214 (19.5)	309 (28.2)	267 (24.4)	127 (11.6)
Oral health problems in patients with COPD are often misattributed to ageing.	167 (15.3)	224 (20.5)	316 (28.9)	285 (26.0)	103 (9.4)
Oral health management requires collaboration between medical and dental professionals.	177 (16.2)	211 (19.3)	312 (28.5)	240 (21.9)	155 (14.2)
Oral health assessment is not routinely performed in patients with COPD.	177 (16.2)	204 (18.6)	296 (27.0)	281 (25.7)	137 (12.5)

Data are presented as *n* (%). Percentages may not total exactly 100% because of rounding.

COPD, chronic obstructive pulmonary disease; n, number.

### 3.3 Physicians' confidence in oral health assessment and management

Physicians demonstrated moderate confidence in managing oral health among patients with COPD (mean confidence score: 2.95 ± 0.71). Across all domains, responses were concentrated in the “Somewhat confident” and “Fairly confident” categories, whereas relatively few physicians reported being completely confident (6.4%–12.8%). The highest proportion of complete confidence was observed for documenting oral health findings during COPD care (12.8%), while the lowest was for recognising oral health issues in patients with COPD (6.4%) (Table 3).

**Table 3:** Physicians' confidence in managing oral health in patients with chronic obstructive pulmonary disease (COPD).

Statement	Not confident *n* (%)	Slightly confident *n* (%)	Somewhat confident *n* (%)	Fairly confident *n* (%)	Completely confident *n* (%)
I am confident in recognising oral health issues in patients with COPD.	252 (23.0)	300 (27.4)	284 (25.9)	189 (17.3)	70 (6.4)
I am confident in discussing oral health concerns with patients who have COPD.	159 (14.5)	240 (21.9)	334 (30.5)	246 (22.5)	116 (10.6)
I am confident in providing oral health advice during COPD care.	125 (11.4)	220 (20.1)	348 (31.8)	266 (24.3)	136 (12.4)
I am confident in interpreting oral health findings within the context of COPD management.	130 (11.9)	258 (23.6)	345 (31.5)	251 (22.9)	111 (10.1)
I am confident in promoting the integration of oral health into COPD care protocols.	150 (13.7)	265 (24.2)	343 (31.3)	232 (21.2)	105 (9.6)
I am confident in collaborating with dental professionals in the care of patients with COPD.	128 (11.7)	247 (22.6)	355 (32.4)	234 (21.4)	131 (12.0)
I am confident in assessing the need for oral health screening in patients with COPD.	121 (11.1)	237 (21.6)	361 (33.0)	265 (24.2)	111 (10.1)
I am confident in documenting oral health findings during COPD care.	125 (11.4)	199 (18.2)	309 (28.2)	322 (29.4)	140 (12.8)

Data are presented as *n* (%). Percentages may not total exactly 100% because of rounding.

COPD, chronic obstructive pulmonary disease; n, number.

### 3.4 Current oral health assessment and management practices

Routine oral health practices were inconsistently implemented. Among all physicians, 24.7% reported often or always assessing oral health, 30.0% often or always enquired about oral hygiene practices, and 29.8% often or always consulted or collaborated with dental professionals. Among the 648 physicians who assessed oral health at least sometimes, the most frequently reported action following identification of poor oral health was documentation in the medical record (45.2%), followed by providing basic oral health education (33.2%) and monitoring the issue over time (31.2%). In contrast, 10.0% reported taking no specific action (Table 4).

**Table 4:** Physicians' current oral health practices and actions taken when poor oral health is identified in patients with COPD.

Practice item	Never	Rarely	Sometimes	Often	Always	Yes
Routine oral health practices						
Routinely assess oral health in patients with COPD	186 (17.0)	261 (23.8)	378 (34.5)	181 (16.5)	89 (8.1)	—
Ask patients with COPD about their oral hygiene practices	138 (12.6)	265 (24.2)	364 (33.2)	229 (20.9)	99 (9.0)	—
Consult or collaborate with dental professionals when managing patients with COPD	134 (12.2)	283 (25.8)	352 (32.1)	235 (21.5)	91 (8.3)	—
Actions taken when poor oral health is identified*						
Consult dental professionals	—	—	—	—	—	128 (19.8)
Provide basic oral health education	—	—	—	—	—	215 (33.2)
Document oral health status in the medical record	—	—	—	—	—	293 (45.2)
Monitor the issue over time	—	—	—	—	—	202 (31.2)
Provide direct oral care (e.g., bedside cleaning or antiseptic rinse)	—	—	—	—	—	143 (22.1)
Take no specific action	—	—	—	—	—	65 (10.0)

Data are presented as *n* (%). Percentages may not total exactly 100% because of rounding.

*Multiple responses were permitted for actions taken when poor oral health was identified; this subsection was completed only by physicians who reported assessing oral health sometimes, often, or always (*n* = 648).

COPD, chronic obstructive pulmonary disease; n, number.

### 3.5 Perceived barriers to integrating oral health into COPD care

Several barriers to integrating oral health into COPD care were identified by the participating physicians. The most frequently reported barriers were lack of formal training on the relationship between oral health and COPD (38.5%), insufficient time to assess oral health (28.1%), uncertainty regarding whether oral health assessment is part of the physician's professional role (24.1%), and the absence of workplace protocols (22.6%). Other commonly reported barriers included limited access to dental professionals (19.6%) and lack of confidence in identifying and interpreting oral health issues (16.3%) (Table 5).

**Table 5:** Perceived barriers to integrating oral health into the management of patients with COPD among physicians.

Perceived barrier*	Yes, *n* (%)
No formal training on the relationship between oral health and COPD	421 (38.5)
Insufficient time to assess oral health	308 (28.1)
Uncertainty whether oral health assessment is part of the professional role	264 (24.1)
No workplace protocol for integrating oral health into COPD care	248 (22.6)
Limited access to dental professionals	215 (19.6)
Lack of confidence in identifying and interpreting oral health issues	178 (16.3)
No clear referral pathways between medical and dental services	177 (16.2)
Oral health is not prioritised in COPD-related continuing education	166 (15.2)
Patients with COPD rarely report oral health problems	160 (14.6)
Insufficient resources to address oral health in patients with COPD	141 (12.9)

Data are presented as *n* (%).

*Multiple responses were permitted; therefore, respondents could select more than one perceived barrier and percentages do not sum to 100%.

COPD, chronic obstructive pulmonary disease; n, number.

### 3.6 Factors associated with routine oral health assessment

The attitude and confidence composite scores were moderately positively correlated (r = 0.455, *P* < 0.001). Multicollinearity diagnostics showed low VIF values for attitude (VIF = 1.68) and confidence (VIF = 1.60), with a mean VIF of 1.73 for the multivariable model, indicating no evidence of problematic multicollinearity.

Univariable logistic regression analysis identified several factors associated with routine oral health assessment among physicians caring for patients with COPD (Table 6). Compared with General Practitioners, pulmonologists had significantly higher odds of routinely assessing oral health (OR = 1.83, 95% CI: 1.27–2.64; *P* = 0.001), whereas no significant difference was observed for Critical Care Physicians (OR = 1.25, 95% CI: 0.89–1.73). Greater clinical experience, managing a higher monthly volume of patients with COPD, and higher attitude and confidence scores were also significantly associated with routine oral health assessment in the univariable analysis. In the multivariable analysis, physician specialty, attitude score, and confidence score remained independently associated with routine oral health assessment. Compared with General Practitioners, pulmonologists had significantly higher odds of routinely assessing oral health (adjusted OR = 1.65, 95% CI: 1.11–2.45; *P* = 0.013), whereas Critical Care Physicians did not differ significantly from General Practitioners (adjusted OR = 0.99, 95% CI: 0.69–1.42; *P* = 0.954). Higher attitude (adjusted OR = 1.31, 95% CI: 1.04–1.64; *P* = 0.022) and confidence scores (adjusted OR = 3.00, 95% CI: 2.26–3.99; *P* < 0.001) were also independently associated with the outcome, whereas age, gender, primary workplace, clinical experience, monthly COPD caseload, and previous formal training in oral health care were not.

**Table 6:** Univariable and multivariable logistic regression analyses of factors associated with routine oral health assessment among physicians caring for patients with COPD.

Variable	Univariable OR (95% CI)	*P*-value	Adjusted OR (95% CI)	*P*-value
Age group (years)				
24–30	Reference	—	Reference	—
31–35	1.11 (0.78–1.59)	0.555	0.98 (0.68–1.42)	0.917
36–40	1.00 (0.66–1.52)	0.999	0.96 (0.63–1.47)	0.855
>40	1.02 (0.62–1.69)	0.930	0.86 (0.49–1.52)	0.603
Gender		0.913		0.648
Female	Reference	—	Reference	—
Male	0.98 (0.73–1.32)	0.913	1.07 (0.79–1.46)	0.648
Physician specialty		0.001		0.015
Critical Care Physician	1.25 (0.89–1.73)	0.194	0.99 (0.69–1.42)	0.954
Pulmonologist	1.83 (1.27–2.64)	0.001	1.65 (1.11–2.45)	0.013
General Practitioner	Reference	—	Reference	—
Primary workplace				0.274
Government hospital	0.90 (0.67–1.19)	0.450	0.84 (0.62–1.15)	0.274
Private hospital	Reference	—	Reference	—
Years of clinical experience				
Less than 1 year	Reference	—	Reference	—
1–5 years	1.67 (0.98–2.86)	0.061	0.98 (0.55–1.75)	0.936
6–10 years	2.19 (1.31–3.66)	0.003	1.40 (0.80–2.47)	0.244
More than 10 years	2.27 (1.32–3.89)	0.003	1.31 (0.71–2.44)	0.389
Patients with COPD managed per month				
<10	Reference	—	Reference	—
10–30	1.42 (0.92–2.21)	0.112	1.21 (0.75–1.98)	0.434
31–50	1.56 (1.01–2.41)	0.045	1.14 (0.69–1.87)	0.617
>50	1.79 (1.08–2.98)	0.025	1.04 (0.58–1.89)	0.886
Formal training in oral health care for patients with COPD		0.481		0.394
No	Reference	—	Reference	—
Yes	1.11 (0.83–1.48)	0.481	1.14 (0.84–1.56)	0.394
Attitude score (per one-point increase)	2.00 (1.66–2.42)	<0.001	1.31 (1.04–1.64)	0.022
Confidence score (per one-point increase)	3.42 (2.66–4.39)	<0.001	3.00 (2.26–3.99)	<0.001

Outcome: Routine oral health assessment was defined as reporting “Often” or “Always” assessing oral health in patients with COPD, compared with “Never,” “Rarely,” or “Sometimes.”.

Univariable odds ratios were estimated using separate logistic regression models for each predictor.

Adjusted odds ratios were obtained from a multivariable logistic regression model including age group, gender, physician specialty, primary workplace, years of clinical experience, number of patients with COPD managed per month, formal training in oral health care for patients with COPD, attitude score, and confidence score.

Continuous variables (attitude and confidence scores) are presented as the change in odds associated with a one-point increase in score. Reference categories are indicated in the table.

CI, confidence interval; COPD, chronic obstructive pulmonary disease; OR, odds ratio.

## 4 Discussion

To the best of our knowledge, this is the first study in Saudi Arabia to evaluate physicians’ oral health practices related to the care of patients with COPD and to identify factors associated with the integration of oral health into routine respiratory care. Overall, physicians’ attitudes toward the importance of oral health in COPD management were around the midpoint of the scale, although their confidence in performing oral health-related activities was generally modest. Routine oral health assessment was infrequently incorporated into clinical practice, while patient education and documentation were performed more often than referrals to dental services or the provision of direct oral health care. The most commonly reported barriers were inadequate training and limited time for oral health assessment. Importantly, greater confidence and higher attitude scores were significantly associated with routine oral health assessment in patients with COPD. These findings highlight important gaps in current practice and emphasize the need for targeted educational initiatives and organizational support to better integrate oral health into comprehensive COPD management.

Physicians’ attitudes toward integrating oral health into COPD care were around the midpoint of the scale, while their confidence in performing oral health-related activities remained relatively low. This discrepancy likely reflects growing awareness of the importance of oral health, despite limited education and practical training in oral health assessment during medical education (20). Comparable findings have been reported in diabetes care, where physicians recognize the importance of oral health but infrequently perform oral examinations or refer patients to dental services because of limited knowledge and training (21–23). Likewise, studies in cardiovascular care have shown that cardiologists are not routinely promoting oral health to their patients due to a lack of resources and training, time constraints, and the absence of referral pathways (24, 25). Similar patterns have also been identified in critical care settings, where healthcare professionals recognize the importance of oral care but often fail to provide it consistently because of inadequate knowledge, limited clinical training, the absence of standardized oral care protocols, and insufficient access to specialized oral care tools (26–28). Furthermore, evidence from interprofessional oral health research further demonstrates that educational and collaborative training programs improve healthcare professionals' confidence and promote the integration of oral health into chronic disease management (29, 30). Physician confidence showed the strongest association with routine oral health assessment in our study, highlighting the relationship between greater confidence and more frequent routine oral health assessment (31).

Routine oral health assessment was uncommon among physicians in our study, despite growing evidence that poor oral health is associated with more frequent exacerbations, hospitalisations, and poorer quality of life among patients with COPD (10, 31, 32). Documentation was the most commonly reported oral health practice, possibly because it is easily integrated into routine care. In contrast, referral to dental professionals was the least frequently reported active management action, which may reflect limited access to dental professionals, unclear referral pathways, and uncertainty regarding professional responsibility. However, a previous qualitative study in Brazil showed that although healthcare professionals recognise poor oral health in patients with COPD, comprehensive oral health assessment and referral practices remain limited (4). Collaboration with dental professionals was also limited, reflecting the persistent separation between medical and dental services, which continues to hinder coordinated patient care and timely referral pathways (17, 33). Furthermore, only one-third of physicians reported providing routine oral health education, suggesting that preventive oral care remains insufficiently integrated into COPD management, potentially due to limited confidence and inadequate training in delivering oral health advice (20, 34). These practice gaps may be attributable to unclear referral pathways between physicians and dental professionals, limited availability of oral care supplies, and the absence of oral health care protocols (26, 35). These observations support the need to integrate standardised oral health screening, structured referral systems, and stronger medical-dental collaboration into routine COPD care to facilitate more comprehensive patient management.

Pulmonologists were more likely than General Practitioners to perform routine oral health assessments. This may reflect their dedicated specialty, which provides greater exposure to the long-term management of COPD, fostering greater familiarity with disease-related comorbidities and promoting more comprehensive patient care (36, 37). Although greater clinical experience and managing a higher volume of patients with COPD were associated with routine oral health assessment in the univariable analysis, these associations were no longer significant after adjustment for physician specialty and other covariates. This suggests that the observed associations may partly reflect differences in physician characteristics, particularly specialty, attitudes, and confidence, rather than clinical experience or patient volume alone. Notably, confidence and attitude scores remained independently associated with routine assessment, with higher scores associated with a greater likelihood of reporting routine oral health assessment (38, 39).

The principal barriers to integrating oral health into COPD care were the lack of training, time constraints, the absence of standardized oral health protocols, and uncertainty regarding physicians' role in oral health care. Interestingly, previous formal training was not significantly associated with routine oral health assessment, despite lack of training being the most frequently reported barrier. This may suggest that exposure to training alone does not necessarily translate into routine clinical practice and that the content and practical focus of training may also be important. However, these aspects of training were not assessed in the present study. These barriers may be interconnected, as inadequate training may coexist with lower knowledge and confidence and less frequent oral health assessment, education, and referral practices (26, 35). This pattern is consistent with the broader oral health literature, where lack of training has been identified as a major obstacle to integrating oral health into routine medical practice (39, 40). Time constraints have also been consistently reported as one of the most important barriers, particularly in diabetes management where competing clinical priorities often limit opportunities for preventive oral health care (21, 41). The absence of oral health protocol and clear referral pathways further discourages physicians from incorporating oral health into routine consultations, despite recognizing its clinical importance (42, 43). Role uncertainty represents another important challenge, as many physicians continue to perceive oral health as primarily the responsibility of dental professionals rather than a shared component of comprehensive patient care (44). Comparable barriers have also been reported in critical care settings, where inadequate education, inconsistent protocols, and limited access to oral care resources contribute to suboptimal oral health practices (45, 46). Addressing these obstacles through targeted training, standardized protocols, and integrated medical-dental care pathways may help support the routine incorporation of oral health into COPD management.

The lack of association between previous formal training and routine oral health assessment suggests that existing training programs may not have effectively changed clinical behaviours**.** This finding indicates that the quality and practical connt of training may be more important than training exposure alone. Saudi studies have similarly identified limited oral health competencies among physicians and gaps between oral health knowledge and interdisciplinary practice, supporting greater integration of oral health and interprofessional education into medical training (39, 47–49). The particularly low referral rate may also reflect the separation between medical and dental services and uncertainty regarding physicians’ responsibility for oral health, which may reinforce traditional professional boundaries. Future studies should therefore evaluate the content, practical components, supervision, and reinforcement of oral health training and explore how organisational structures and medical–dental referral pathways influence its integration into routine COPD care.

The findings of this study have several important clinical implications. Oral health should be recognised as an integral component of comprehensive COPD management, given the growing evidence linking poor oral health with adverse respiratory outcomes (50, 51). Integrating oral health into undergraduate medical education may help equip future physicians with the knowledge and skills required to identify common oral conditions and understand their impact on systemic health (52–54). In addition, continuing professional development programs may provide opportunities for practical training in oral health assessment, patient education, and appropriate referral practices (55). The development of standardised clinical pathways and well-defined referral systems between respiratory and dental services may facilitate early identification and management of oral health problems while improving continuity of care (56). Strengthening interprofessional collaboration between physicians, dentists, and other healthcare professionals may also support coordinated and comprehensive patient care (57, 58). Furthermore, incorporating oral health screening prompts, documentation templates, and referral reminders into electronic medical records may be a means of supporting oral health assessment within routine clinical workflows (59). However, these proposed strategies were not evaluated in the present study, and their effectiveness requires further investigation. Future COPD guidelines may consider recommendations on oral health screening, referral pathways, and interdisciplinary collaboration to support the integration of oral health into routine clinical practice.

### 4.1 Strengths and limitations

This study is noteworthy as the first in Saudi Arabia to evaluate physicians’ oral health practices in COPD and identify factors independently associated with routine oral health assessment. It included a large national sample of physicians across multiple specialities involved in COPD care and utilised a comprehensive questionnaire that assessed key domains, including oral health attitudes, confidence, clinical practices, and perceived barriers. Nevertheless, several limitations should be acknowledged. Given the cross-sectional design, the observed relationships should be interpreted as associations rather than causal relationships, and the temporal sequence between physician characteristics and oral health practices cannot be established. Longitudinal studies are needed to further examine the temporal nature of these associations. The assessment of clinical practices was based on self-reported data, which may have introduced recall bias and social desirability bias. In addition, both the primary outcome and the attitude and confidence scores were self-reported within the same questionnaire, which may have introduced shared-method variance and potentially inflated the observed associations. The use of convenience sampling may have introduced selection bias and limited the sample's representativeness. Recruitment through social messaging and online platforms may also have preferentially reached younger or more digitally engaged physicians. Furthermore, because the survey was disseminated through multiple professional networks and social media platforms, with the possibility of onward forwarding, the total number of eligible physicians exposed to the survey invitation could not be determined, precluding calculation of a conventional response rate. The findings may have limited generalizability beyond Saudi Arabia due to differences in healthcare systems, medical training, and clinical practice environments. Future interventional studies are needed to determine whether targeted educational programs and structured clinical pathways can improve the integration of oral health assessment into COPD management.

## 5 Conclusion

This study provides the first evidence from Saudi Arabia regarding physicians' oral health practices in COPD care and highlights important gaps in the routine integration of oral health assessment. Although physicians recognized the importance of oral health, confidence and clinical implementation remained limited, with training gaps and practical barriers affecting practice. Higher confidence and attitude scores were independently associated with routine oral health assessment, highlighting their potential relevance to the integration of oral health into COPD care. Given the cross-sectional design, these associations should not be interpreted as evidence of causal relationships. Further interventional studies are needed to evaluate effective strategies for incorporating oral health care into routine COPD management.

## Data Availability

The data supporting the findings of this study are available from the corresponding author upon reasonable request.
